# Computer-assisted counting of retinal cells by automatic segmentation after TV denoising

**DOI:** 10.1186/1471-2415-13-59

**Published:** 2013-10-20

**Authors:** Kristian Bredies, Marcus Wagner, Christian Schubert, Peter Ahnelt

**Affiliations:** 1 Institute for Mathematics and Scientific Computing, University of Graz, Heinrichstraße 36, Graz, A-8010, Austria; 2 Department of Mathematics, University of Leipzig, P. O. B. 10 09 20, Leipzig, D-04009, Germany; 3 Center for Physiology and Pharmacology, Medical University Vienna, Schwarzspanierstraße 17, Wien, A-1090, Austria

**Keywords:** Mammalian photoreceptor cells, Automatical counting, Adaptive algorithm, Continuous optimization, Total variation denoising

## Abstract

**Background:**

Quantitative evaluation of mosaics of photoreceptors and neurons is essential in studies on development, aging and degeneration of the retina. Manual counting of samples is a time consuming procedure while attempts to automatization are subject to various restrictions from biological and preparation variability leading to both over- and underestimation of cell numbers. Here we present an adaptive algorithm to overcome many of these problems.

Digital micrographs were obtained from cone photoreceptor mosaics visualized by anti-opsin immuno-cytochemistry in retinal wholemounts from a variety of mammalian species including primates. Segmentation of photoreceptors (from background, debris, blood vessels, other cell types) was performed by a procedure based on Rudin-Osher-Fatemi total variation (TV) denoising. Once 3 parameters are manually adjusted based on a sample, similarly structured images can be batch processed. The module is implemented in MATLAB and fully documented online.

**Results:**

The object recognition procedure was tested on samples with a typical range of signal and background variations. We obtained results with error ratios of less than 10% in 16 of 18 samples and a mean error of less than 6% compared to manual counts.

**Conclusions:**

The presented method provides a traceable module for automated acquisition of retinal cell density data. Remaining errors, including addition of background items, splitting or merging of objects might be further reduced by introduction of additional parameters. The module may be integrated into extended environments with features such as 3D-acquisition and recognition.

## Background

### Introduction

The vertebrate retina contains two or more subtypes of photoreceptors and dozens of interneuron types, thus being organized for effective operation at different light levels and at different bands of the sunlight’s spectrum. Regional shifts in densities and proportions of the subtypes of photoreceptors and interneurons in the retina have been studied intensively as they are assumed to reflect both the evolution of species and specific adaptations to their lifestyle [1,2]. Deviations within cell density and mosaic regularity from their normal variability are of specific interest for research involved in developmental control or progressive loss of photoreceptor and other cells due to degenerative diseases and other pathologic processes in the visual system.

Obtaining such data implies the acquisition and analysis of a large number of samples, which is often a time-consuming task requiring persons with appropriate training and experience. Given, moreover, the approximately planar organization of retinal layers, the problem of detection and counting of photoreceptor cells is a promising candidate for at least partial automatization. However, while various papers have developed options for mapping and analysis of retinal cell mosaic data, once they are digitized [3-7], approaches to full automatization of the time-consuming procedure of actual identification and counting have been rare (we mention [8-10]). Besides the time and computing power required for acquiring two- and three-dimensional representations of the tissue of interest, the heterogeneity of the tissue itself is still a major challenge. Many parameters such as tissue thickness/transparency, preparatory and manipulatory distortions change across the retinas, and the cells of interest themselves change in size, shape and spacing. Consequently, for reliable detection of the targets and their differentiation from other items such as debris, other cells, local damage or blood vessels highly adaptive algorithms are required.

The present approach focuses on addressing these problems for the segmentation and counting of photoreceptors. We propose an automatic detection and counting procedure, which is based on Rudin-Osher-Fatemi total variation (TV) denoising [11] with subsequent segmentation. As the comparison with manually collected data shows, this method is able to detect the targets at comparable reliability. In the view of the authors, the main advantage of the method consists in the complete traceability of all data processing steps and its reproducibility independently from a particular software platform. Thus a possibility for standardization and direct comparisons of automatic counts for samples obtained within different environments is provided.

In the present study, the method has been implemented as a MATLAB module and has been applied to single frames (and montages). The method, however, is not limited to the processing of two-dimensional data and can be equally implemented for the analysis of three- or multidimensional data stacks. It could as well be integrated into more comprehensive motorized acquisition setups for stereological sampling or complete mapping of retinal populations.

### Retinal image data

**Figure 1:**
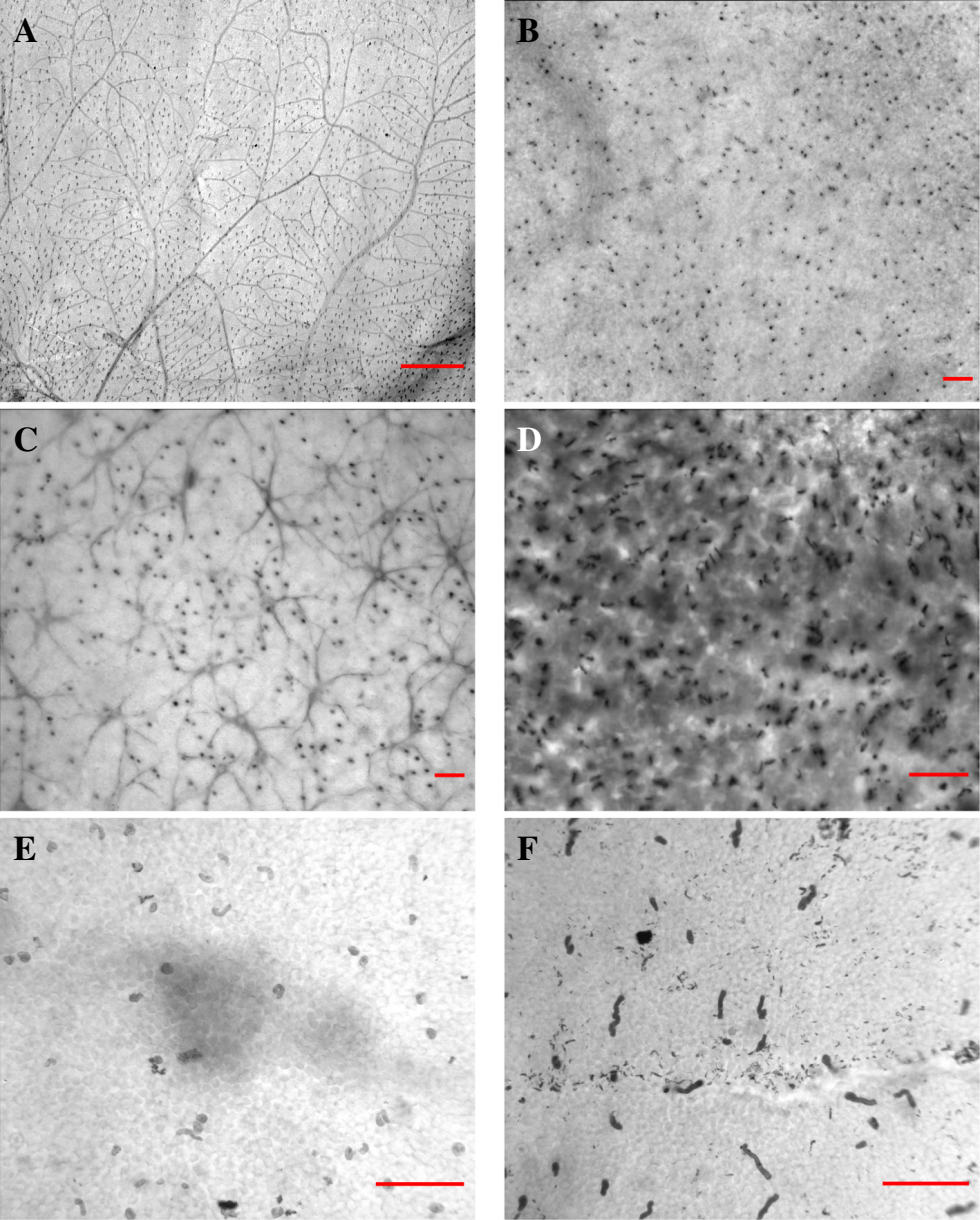
**Examples of retinal micrographs used in the experiments.** The scale, indicated by the red bar, is 500 *μ*m in (A) and 50 *μ*m in (B)–(F). **(A)** Orangutan, No. 1, S-cones and vessels labeled. **(B)** Domestic cat, No. 4 (clip), S-cones labeled. **(C)** Jaguar, No. 11 (clip), S-cones and horizontal cells labeled. **(D)** Pangolin, No. 12 (clip), M-/L-cones labeled. **(E)** Agouti, No. 13, S-cones labeled. **(F)** Agouti, No. 16, S-cones labeled. For more details, see Table 1.

Retinal samples from the following species have been used: Orangutan (*Pongo pygmaeus* (L. 1760)), Domestic cat (*Felis silvestris catus* (L. 1758)), Manul (*Felis manul* (Pallas 1776)), Eurasian Lynx (*Lynx lynx* (L. 1758)), Cheetah (*Acinonyx jubatus* (Schreber 1775)), Jaguar (*Panthera onca* (L. 1758)), Long-tailed Pangolin (*Manis tetradactyla* (L. 1766)) and Black-rumped Agouti (*Dasyprocta prymnolopha* (Wagler 1831)) (see Table 1 and Figure 1).

**Table 1:** Retinal image data

**No.**	**Species**	**Eye**	**Retina region**	**Labeled structures**	**Staining**	**Density per mm^**2**^**	**File name**	**Image size**	**Remarks about image quality and particularities**	**Shown in Figure(s)**
1	Orangutan	Left	Sup. periphery,	S-cones,	JH455/DAB,	190 (cones)	OrangWhM	1024 ×854	Sufficient quality,	1A
			between 10 mm from	blood vessels	Collagen IV/DAB				but vessels present	
			fovea and ora serrata							
2	Orangutan	Left	Inf. periphery,	S-cones,	JH455/DAB,	156 (cones)	OrUinf-0001pr	1026 ×854	Sufficient quality,	4I–J
			between 11 mm from	blood vessels	Collagen IV/DAB				but vessels present	
			fovea and ora serrata							
3	Domestic cat			S-cones	JH455/DAB	860	loStack	1024 ×1024	Sufficient quality and contrast,	
									but small cones	
4	Domestic cat		Inf. retina	S-cones	JH455/DAB	851	luStack	1024 ×1024	Sufficient quality and contrast,	1B
									but small cones	
5	Domestic cat			S-cones	JH455/DAB	827	roStack	1024 ×1024	Sufficient quality and contrast,	
									but small cones	
6	Domestic cat			S-cones	JH455/DAB	904	ruStack	1024 ×1024	Sufficient quality and contrast,	
									but small cones	
7	Manul			S-cones	JH455/DAB	195	bcergcomp	1024 ×1024	Sufficient quality and contrast,	
									but small cones	
8	Eurasian Lynx	Left	Periphery	S-cones	JH455/DAB	1076	Movie-17-1	1024 ×1024	Good quality and contrast	4A–B
9	Cheetah			S-cones	JH455/DAB	130	1+5CSCStack	1024 ×1024	Good quality and contrast	
10	Cheetah			S-cones	JH455/DAB	1055	Stack SCFoc.	1024 ×1024	Good quality and contrast	
11	Jaguar	Right	Periphery	S-cones,	JH455/DAB	440 (cones)	Combination	1024 ×1024	Good quality, but additional	1C, 4G–H
				horizontal cells					cell type present	
12	Pangolin	Right	Mid periphery	M-/L-cones	JH492/DAB	2290	492_20_09	1024 ×1024	Background very blotchy	1D, 2A–F,
										4C–D
13	Agouti	Left	Sup. temp.	S-cones	JH455/DAB	705	A4scal	1024 ×853	Low contrast, cell debris present	1E
			periphery							
14	Agouti			S-cones	JH455/DAB	790	A5scal	1024 ×853	Low contrast, cell debris present	
15	Agouti			S-cones	JH455/DAB	855	A6scal	1024 ×853	Low contrast, cell debris present	
16	Agouti	left	Temp. periphery	S-cones	JH455/DAB	480	B1scal	1024 ×853	Poor quality, cell debris present	1F, 4E–F
17	Agouti			S-cones	JH455/DAB	905	B2scal	1024 ×853	Poor quality, cell debris present	
18	Agouti			S-cones	JH455/DAB	3500	C4scal	1024 ×853	Poor quality, cell debris present	

Most of the eyes were obtained from animals delivered to veterinary pathology from zoos and animal parks; some originate from collaborations for other studies [12,13]. Post mortem times were between 0.5 and 12 hours. Eyes were enucleated and immersed in 0.01M phosphate buffer saline (PBS, pH 7.4) with 4% paraformaldehyde. Some were treated after being opened with a cut along the corneal limbus for faster penetration of fixative. Retina wholemounts were prepared in PBS and flattened by radial cuts, in order to preserve the horizontal and vertical meridian. Cone photoreceptors sensitive to medium/long wavelengths (M-/L-cones) were labeled in isolated Pangolin retina using JH492 antibody, in all other retinas S-cones were labeled using JH455 (both antibodies provided by J. Nathans [14]). In peripheral Jaguar retina, in addition to S-cones horizontal cells are (unintentionally) co-labeled by JH455. Retinal vessels in Orangutan were labeled with rabbit anti-mouse collagen IV (AbD Serotec, 2150-1470). After incubation in primary antisera overnight for up to 3 days visualization was done using goat anti-rabbit igg-biotin (Sigma, B7389), ExtrAvidin-peroxidase conjugate (Sigma, E2886) and the diaminobenzidine (DAB) reaction. After washing in PBS, retinas were gradually transferred up to 90% glycerol, mounted with photoreceptor-side up on a glass slide, and cover slipped.

Manual counting of labeled cones was done within sampling frames of 150×150*μ*m or 300×300*μ*m using an online-video overlay system consisting of Canvas 5 (ACD Systems, USA) software on a Macintosh computer connected with a Hamamatsu 2400 analog camera attached to a Nikon Eclipse microscope. This system allows dual live view of the specimen: through the microscope’s optics or on the video image overlaid by the sampling frames. Optional change of focus and illumination supports optimized online identification of cells and exclusion of artifacts by position, form, color and other details.

The images (8 or 10 bit grey scale) used for computer-assisted cell counting were obtained by using a Photometrix Camera model CH250/A connected to a Nikon Eclipse E600 microscope (magnification factors 200× to 600×) using QED Imaging Software (QED Imaging Inc., Pittsburgh, PA) on a Macintosh computer. In most cases, a projection image (max density or sum) was composed from a stack of images at relevant focus levels using the public domain NIH Image program (developed at the U.S. National Institutes of Health; available at http://rsb.info.nih.gov/nih-image/ (accessed 11.02. 2013)).

For testing the computer-assisted cell counting method, images were chosen by the following criteria: retinas from different species, differences in quality concerning morphology (different amount of cell debris, broken cones etc.) and contrast between labeled cells and background (see Table 1). Images with additional labeled structures like horizontal cells or blood vessels have been analyzed as well. The respective objects of interest were then manually counted by an experienced coauthor (C.S.), and the results were tabulated for comparison with the outcome from the automatic counting method (see below).

### Description of the detection and counting method

**Figure 2:**
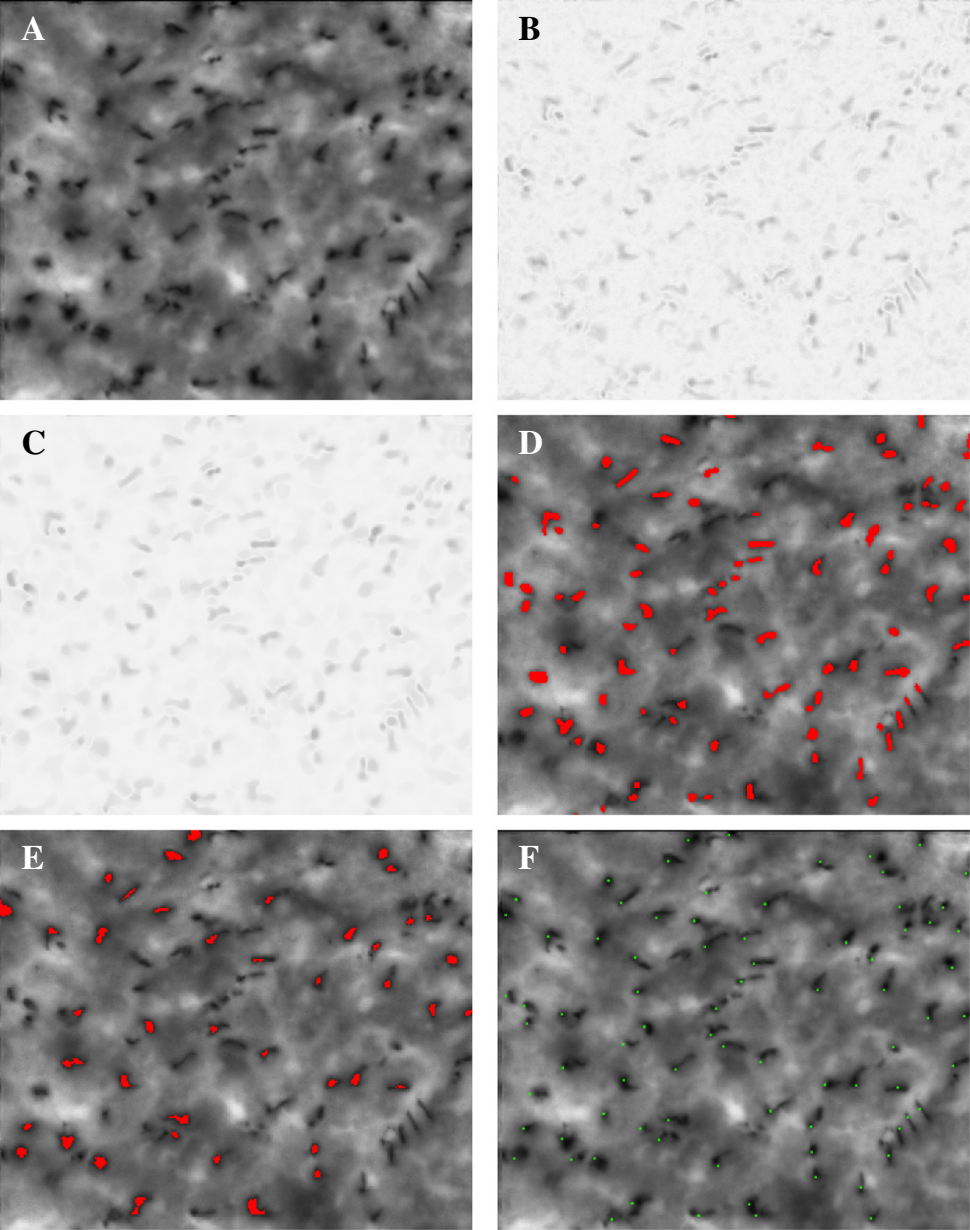
**The counting procedure.(A)** Original data (Pangolin, No. 12, upper left part). **(B)** Output after Step 1 (subtraction of median), grey values divided by factor 1.05. **(C)** Output of TV denoising after Step 2, grey values divided by factor 1.05. **(D)** Output of Step 4, superimposed to the original image (counted features dyed in red color). **(E)** Result of direct segmentation, superimposed to the original image (counted features dyed in red color). **(F)** Result of manual count (counted cells tagged with green dots).

Due to the reasons mentioned in the introduction, the immediate segmentation of the retina image data *I*^(0)^ by intensity thresholding leads in many instances to poor results, see below. Therefore, we carry out two processing steps before segmentation. In a first step, we generate from the original image *I*^(0)^, cf. Figure 2A, a median-filtered version $\tilde{I}$ using window size *m* and subtract it from *I*^(0)^, which results in a considerable removal of brightness fluctuations in the retinal background, cf. Figure 2B. Subsequently, we subject the image $I^{\left(0\right)}-\tilde{I}=I^{\left(1\right)}$ to a Rudin-Osher-Fatemi TV denoising procedure, cf. [11]. This method, representing a well-established standard in mathematical image processing, may be understood as a kind of filtering, which generates a coarsened, cartoon-like version of the input data, cf. Figure 2C. Nevertheless, during this procedure the images of the dyed retinal cells will be conserved as spots. In mathematical terms, TV denoising means to solve an continuous optimization problem, namely

(1) $$F(x)\;=\;\int_{\Omega }{\left(\;x(s)-I^{\left(1\right)}(s)\;\right)}^{2}\;\text{ds}+\frac{\alpha }{2}\;|\;x\;{|}_{\text{TV}}\to \;\text{Min}\;!$$

for an unknown function *x*(*s*) : *Ω* → [ 0, 1 ], which represents the converted (“filtered”) image. For more details, we refer to the Appendix. Let us only remark that the number *α*>0 within (1) remains fixed from the outset. For the numerical solution of problem (1), surprisingly efficient methods are available by now. In our present study, a recently published solver (by Chambolle/Pock, cf. [15]) has been implemented as a MATLAB subroutine. The segmentation of the output *x* = (*x*_*ij*_) of the TV denoising step will be performed now by application of the following rule: After calculating the expectation *E*(*x*) and variance *Var*(*x*), we declare all pixels *x*_*ij*_ with

(2) $$x_{\text{ij}}<E(x)-c\cdot \text{Var}(x)$$

as “black enough” to belong to images of photoreceptor cells. Finally, all “black” features consisting of a number of connected adjacent pixels, which is bigger than a given threshold size *f*, are automatically counted, cf. Figure 2D.

In this method, no more than three parameters remain to be adjusted manually. These are: the window size *m* for the median filter, the parameter *c* in (2), which influences the contrast differentiation between photoreceptor cells and the background, and the minimal size *f* of a connected feature to be recognized as a photoreceptor cell.

Our algorithm can be summarized as follows:

#### Algorithm 1 **Automatic segmentation after TV denoising**

## Implementation

### Implementation as a MATLAB tool

**Figure 3:**
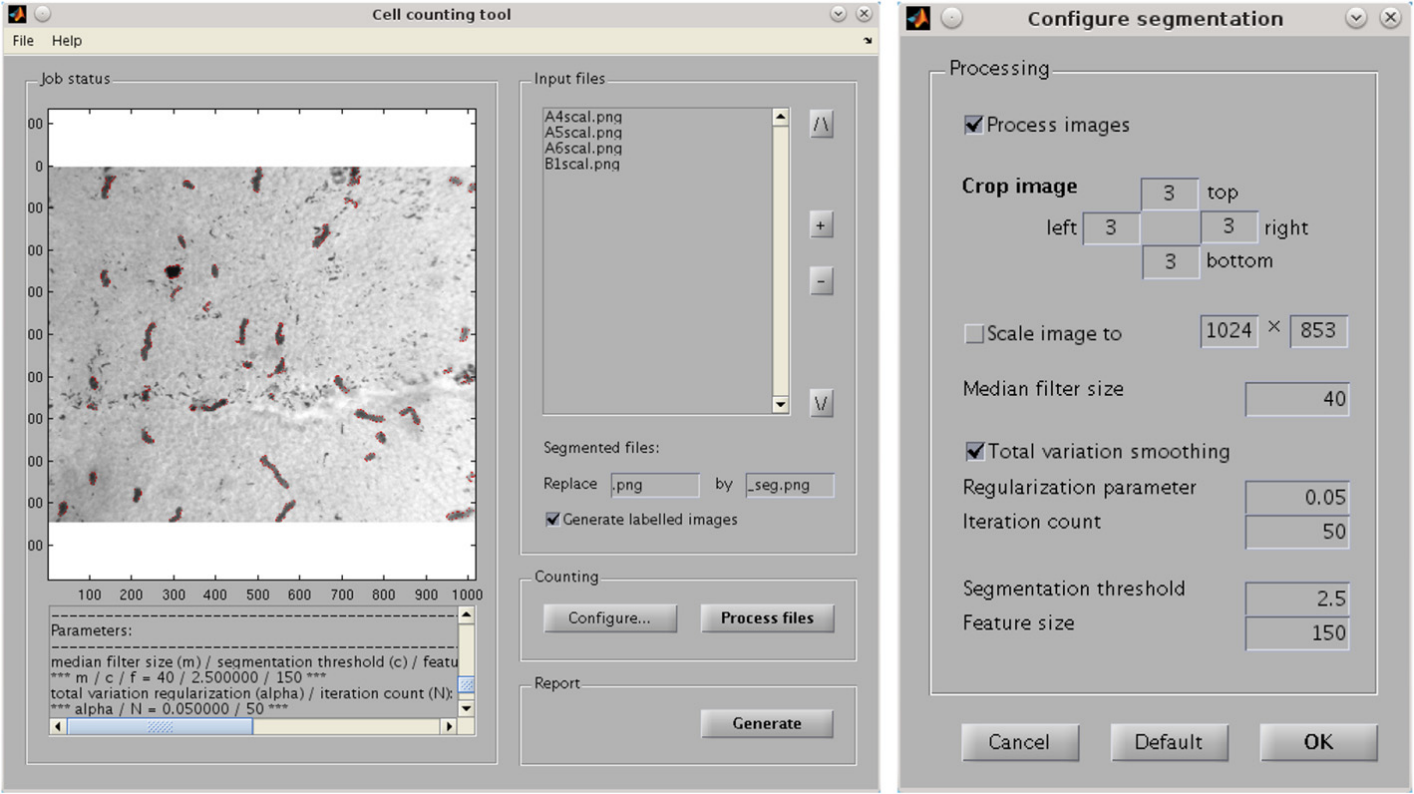
**Screenshots of the MATLAB software tool.(A)** Main graphical user interface. **(B)** The configuration dialog.

Algorithm 1 has been implemented as a MATLAB tool with a graphical user interface (cf. Figure 3A), which allows for batch processing of multiple images. It has been tested on MATLAB 7.14.0.739 (R2012a) and requires the MATLAB Image Processing Toolbox (documented at http://www.mathworks.com/products/matlab and http://www.mathworks.com/products/image (accessed 11.02.2013)). In the following, details regarding the implementation of the procedure are given. In Step 1, the background homogenization, the median filtering is realized in a straightforward manner by calling the MATLAB procedure medfilt2(image, [m,m], ’symmetric’) which is part of the image processing toolbox. For the TV denoising in Step 2, the primal-dual algorithm from [15] is utilized. It is realized by performing *N* steps of the iteration

(3) $$\left\{\begin{array}{l} \;\;\;\left(\begin{array}{l} p^{(1),k+1}\\ p^{(2),k+1} \end{array}\right)=P_{\alpha }\left(\;\left(\begin{array}{l} p^{(1),k}\\ p^{(2),k} \end{array}\right)+\tau_{d}\left(\begin{array}{l} \partial_{s_{1}}^{+}{\bar{x}}^{k}\\ \partial_{s_{2}}^{+}{\bar{x}}^{k} \end{array}\right)\;\right)\;,\;\;P_{\alpha }{\left(v\right)}_{\text{ij}}\\ \;\;\;\;\;\;\;=\frac{\alpha }{\;\text{Max}\;\left(\alpha ,\sqrt{{\left(\;v_{\text{ij}}^{\left(1\right)}\;\right)}^{2}\;+\;{\left(\;v_{\text{ij}}^{\left(2\right)}\;\right)}^{2}}\right)}\;\left(\begin{array}{l} v_{\text{ij}}^{\left(1\right)}\\ v_{\text{ij}}^{\left(2\right)} \end{array}\right)\\ x^{k+1}=\frac{1}{1+\tau_{p}}\;x^{k}\;+\;\frac{\tau_{p}}{1\;+\;\tau_{p}}\;\left(\;I^{\left(1\right)}+\partial_{s_{1}}^{-}p^{(1),k+1}+\partial_{s_{2}}^{-}p^{(2),k+1}\;\right)\\ {\bar{x}}^{k+1}=2x^{k+1}-{\bar{x}}^{k} \end{array}\right)$$

with step sizes *τ*_*p*_,*τ*_*d*_ > 0 such that *τ*_*p*_·*τ*_*d*_ ≤ 0.125 and the forward and backward finite difference operators

(4) $$\begin{array}{ll} {\left(\;\partial_{s_{1}}^{+}{\bar{x}}^{k}\;\right)}_{\text{ij}} & =\left\{\begin{array}{ll} {\bar{x}}_{i+1,j}^{k}- & {\bar{x}}_{\text{ij}}^{k}\;\text{if}\;1\leq i<n\;,\\ & 0\;\text{else}\;, \end{array}\right)\;{\left(\partial_{s_{2}}^{+}{\bar{x}}^{k}\right)}_{\text{ij}}\\ & =\left\{\begin{array}{ll} {\bar{x}}_{i,j+1}^{k}- & {\bar{x}}_{\text{ij}}^{k}\;\text{if}\;1\leq j<r\;,\\ & 0\;\text{else,} \end{array}\right) \end{array}$$

(5) $$\begin{array}{l} \begin{array}{c} {\left(\partial_{s_{1}}^{-}p^{(1),k+1}\;\right)}_{\text{ij}}=\left\{\begin{array}{ll} \;p_{1j}^{(1),k+1}\;\;\;\;\;\; & \text{if}\;i=1\;,\\ p_{\text{ij}}^{(1),k+1}-\;p_{i-1,j}^{(1),k+1}\; & \text{if}\;1<i<n\;,\\ \;-\;p_{n-1,j}^{(1),k+1}\;\; & \text{if}\;i=n\;, \end{array}\right) \end{array} \end{array}$$

(6) $$\begin{array}{c} {\left(\partial_{s_{2}}^{-}p^{(2),k+1}\;\right)}_{\text{ij}}=\left\{\begin{array}{ll} \;p_{i1}^{(2),k+1}\;\;\;\;\;\; & \text{if}\;j=1\;,\\ p_{\text{ij}}^{(2),k+1}-\;p_{i,j-1}^{(2),k+1}\; & \text{if}\;1<j<r\;,\\ \;-\;p_{i,r-1}^{(2),k+1}\;\; & \text{if}\;j=r\;. \end{array}\right) \end{array}$$

The initializations are $x^{0}={\bar{x}}^{0}=I^{\left(1\right)}$, *p*^(1),0^ = *p*^(2),0^ = 0, and the output will be given by $\left(x_{\text{ij}})=(x_{\text{ij}}^{N}\right)$. As default values of the regularization parameter and the number of iterations, we use *α*=0.05 and *N*=50. Note that, in principle, other minimization algorithms could be implemented for solving the TV denoising problem. For instance, we mention the generalized TV approach from [16] and the optimal control method described in [17], which is based on the interior-point solver IPOPT [18,19].

The thresholding in Step 3 is realized with the help of the built-in MATLAB functions mean and var. Finally, for Step 4, the labelling and counting procedure, the function bwlabel is utilized, which is again part of the image processing toolbox. It yields a labelled image in which each connected component is identified by a positive integer. With this information, the identification and counting of those connected components, which comprise at least *f* pixels, can be easily realized.

### Usage

Usually, in order to analyze the topography of items in a retina preparation, a considerable number of image files has to be generated, each showing a segment. Our software tool was especially designed to cope with multiple files showing similar structures. In this situation, it is possible to start with a manual count within one or two typical images in order to calibrate the parameters *m*, *c* and *f*. This can be done by starting the program and adding a single image to the file list. In most cases, the default parameters give a good starting point, hence one can perform a segmentation in order to decide whether one is satisfied with the results. Otherwise, adjust the parameters *m*, *c* and *f* using the configuration dialog (cf. Figure 3B) and try again. Once the parameters have been adjusted, they can be utilized for the analysis of the whole image set. The batch processing feature of the software allows to perform this analysis without further user interaction: simply add the remaining files to the list and start the segmentation procedure. Finally, a report which lists, for each file in the batch, the number and mean density of detected cells as well as their positions, can be automatically generated. As default values for the parameters *m*, *c* and *f*, we encoded *m*=30, *c*=2.5 and *f*=5. Eventually, for convenience and reproducibility, the tool also provides saving and loading of the file list as well as of a list of all parameters.

## Results and discussion

The results of automatical counting are documented in Tables 2, 3 and 4. In order to justify the application of a counting procedure, it is essential that manually counted data are reliably matched. Consequently, the counts generated by TV denoising and subsequent segmentation are compared with carefully realized manual counts. The results are listed in Table 2. Furthermore, for every automatic count the number of recognized cells, which have also been marked in the manual counting procedure (Table 3), as well as the number of artifacts (Table 4) were identified. For comparison, we performed automatic counts by direct segmentation without preceding TV denoising using the same parameters *c* and *f* as in Algorithm 1.

**Table 2:** Overall cell counts by different methods

**No.**	**Image**	**Manual count #**	**Direct segm.**	**TV denoising & segm.**	***m* /*c* /*f***
			**# / rel. error**	**# / rel. error**	
	**Orangutan**				
1	OrangWhM	2328	1784 / 24.9	2286 / 1.8	30 / 2.0 / 5
2	OrUinf-0001pr	1192	793 / 33.5	1168 / 2.0	30 / 3.0 / 5
	**Domestic cat**				
3	loStack	557	518 / 7.0	543 / 2.5	30 / 2.5 / 5
4	luStack	552	584 / 5.8	562 / 1.8	30 / 2.5 / 5
5	roStack	536	539 / 0.6	520 / 3.0	30 / 2.5 / 5
6	ruStack	584	573 / 1.9	573 / 1.9	30 / 2.5 / 5
	**Manul**				
7	bcergcomp	128	110 / 14.1	128 / 0.0	20 / 2.5 / 25
	**Lynx**				
8	Movie-17-1	708	675 / 4.7	659 / 6.9	30 / 2.5 / 5
	**Cheetah**				
9	1+5CSCStack	85	84 / 1.2	91 / 7.1	30 / 3.5 / 40
10	Stack SCFoc.	687	656 / 4.5	634 / 7.7	30 / 2.5 / 5
	**Jaguar**				
11	Combination	289 (S-cones)	177 / 38.8	275 / 4.8	30 / 3.5 / 5
	**Pangolin**				
12	492_20_09	369	109 / 70.5	367 / 0.5	20 / 2.5 / 25
	**Agouti**				
13	A4scal	43	14 / 67.4	44 / 2.3	40 / 2.5 / 150
14	A5scal	48	20 / 58.3	54 / 12.5	40 / 2.5 / 150
15	A6scal	52	44 / 15.4	53 / 1.9	40 / 2.5 / 150
16	B1scal	31	35 / 12.9	43 / 38.7	40 / 2.5 / 150
17	B2scal	55	51 / 7.3	56 / 1.8	40 / 2.5 / 150
18	C4scal	213 (?)	95 / 55.4	234 / 9.8	30 / 2.5 / 50
	**Mean error**		23.6	5.9	
	**Standard dev.**		*23.6*	*8.6*	

Within Algorithm 1, the following parameters have been chosen: The median is taken over a field of *m*×*m* pixels, the TV denoising procedure is applied with regularization parameter *α* = 0.05 and 50 iterations, the contrast differentiation parameter is *c*, and the minimal size of a recognized feature is *f* pixels. For comparison, a direct segmentation of the images has been performed as well, applying Steps 3 and 4 of Algorithm 1 with the same values of *c* and *f* directly to the image data. Together with the automatic counts, their accuracy is given in terms of the relative error (in percents) related to the manual counts from the third column.

**Table 3:** Number of correctly recognized cells within the automatic counts

**No.**	**Image #**	**Manual count**	**Direct segm.**	**TV denoising & segm.**	***m* /*c* /*f***
			**# / percent**	**# / percent**	
	**Orangutan**				
1	OrangWhM	2328	1652 / 71.0	2176 / 93.4	30 / 2.0 / 5
2	OrUinf-0001pr	1192	735 / 61.7	1077 / 90.4	30 / 3.0 / 5
	**Domestic cat**				
3	loStack	557	474 / 85.1	512 / 91.9	30 / 2.5 / 5
4	luStack	552	531 / 96.2	527 / 95.5	30 / 2.5 / 5
5	roStack	536	502 / 93.6	499 / 93.1	30 / 2.5 / 5
6	ruStack	584	530 / 90.8	545 / 93.3	30 / 2.5 / 5
	**Manul**				
7	bcergcomp	128	80 / 62.5	126 / 98.4	20 / 2.5 / 25
	**Lynx**				
8	Movie-17–1	708	642 / 90.7	640 / 90.4	30 / 2.5 / 5
	**Cheetah**				
9	1+5CSCStack	85	81 / 95.3	85 / 100.0	30 / 3.5 / 40
10	Stack SCFoc.	687	637 / 92.7	624 / 90.8	30 / 2.5 / 5
	**Jaguar**				
11	Combination	289 (cones)	164 / 56.7	253 / 87.5	30 / 3.5 / 5
	**Pangolin**				
12	492_20_09	369	104 / 28.2	297 / 80.5	20 / 2.5 / 25
	**Agouti**				
13	A4scal	43	12 / 27.9	40 / 93.0	40 / 2.5 / 150
14	A5scal	48	16 / 33.3	45 / 93.8	40 / 2.5 / 150
15	A6scal	52	40 / 76.9	50 / 96.2	40 / 2.5 / 150
16	B1scal	31	29 / 93.5	31 / 100.0	40 / 2.5 / 150
17	B2scal	55	50 / 90.9	54 / 98.2	40 / 2.5 / 150
18	C4scal	213 (?)	77 / 36.1	185 / 86.8	30 / 2.5 / 50
	**Mean percentage**		71.3	93.0	
	**Standard dev.**		*24.5*	*4.8*	

The percentages are given in relation to the manual counts in the third column.

**Table 4:** Artifacts within the automatic counts produced by the different methods

**No.**	**Image**	**Direct segm.**	**TV denoising & segm.**	***m* /*c* /*f***
		**# / percent**	**# / percent**	
	**Orangutan**			
1	OrangWhM	132 / 7.4	110 / 4.8	30 / 2.0 / 5
2	OrUinf-0001pr	58 / 7.3	91 / 7.8	30 / 3.0 / 5
	**Domestic cat**			
3	loStack	44 / 8.5	31 / 5.7	30 / 2.5 / 5
4	luStack	53 / 9.1	35 / 6.2	30 / 2.5 / 5
5	roStack	37 / 6.9	21 / 4.0	30 / 2.5 / 5
6	ruStack	43 / 7.5	28 / 4.9	30 / 2.5 / 5
	**Manul**			
7	bcergcomp	30 / 23.4	2 / 1.6	20 / 2.5 / 25
	**Lynx**			
8	Movie-17–1	33 / 4.9	19 / 2.9	30 / 2.5 / 5
	**Cheetah**			
9	1+5CSCStack	3 / 3.6	6 / 6.6	30 / 3.5 / 40
10	Stack SCFoc.	19 / 2.9	10 / 1.6	30 / 2.5 / 5
	**Jaguar**			
11	Combination	13 / 7.3	22 / 8.0	30 / 3.5 / 5
	**Pangolin**			
12	492_20_09	5 / 4.6	70 / 19.1	20 / 2.5 / 25
	**Agouti**			
13	A4scal	2 / 14.3	4 / 9.1	40 / 2.5 / 150
14	A5scal	4 / 20.0	9 / 16.7	40 / 2.5 / 150
15	A6scal	4 / 9.1	3 / 5.7	40 / 2.5 / 150
16	B1scal	6 / 17.1	12 / 27.9	40 / 2.5 / 150
17	B2scal	1 / 2.0	2 / 3.6	40 / 2.5 / 150
18	C4scal	18 / 18.9	49 / 20.9	30 / 2.5 / 50
	**Mean percentage**	9.7	8.7	
	**Standard dev.**	*6.1*	*7.2*	

The percentages are given in relation to the corresponding counts from columns 4 and 5 in Table 2.

The quality requirements for automatic counts depend on the specific goal of interest. When assessing general distribution patterns or gradients for certain types of receptors, a relative error of 10% may be acceptable while for the analysis of degeneration or proliferation phenomena or the detection of initial points of density changes, an error of about 5% and less is desirable. Table 2 shows that the latter goal has been realized by the TV/segmentation method in 12 of 18 cases while the error is below 10% in 16 of 18 cases. The mean error amounts to 5.9%. Moreover, Table 3 shows that the method recognizes correctly more than 90% of the manually marked cells in 15 of 18 cases (93.0% in the mean). The number of artifacts contained in the counts, as listed in Table 4, amounts to 8.7% in the mean. Thus, given that the TV/segmentation method makes no use of additional information about the shape of the cells or the variation of their size, these results are quite satisfactory.

As to be expected, our results show that automatic counting by the TV/segmentation method is superior to direct segmentation in every respect. The mean relative error produced by the latter amounts to as much as 23.6%, and the relative error goes below 10% in 8 of 18 cases only. The loss of precision is mostly caused by the fact that, by direct segmentation, a significantly smaller number of marked cells is recognized than by the TV/segmentation method (cf. Figure 2D–F) while the ratio of artifacts produced by both methods is comparable. The superiority of the TV/segmentation method can be seen as well by comparing the standard deviations of the indicators. Let us remark that, during our experiments, we further observed that the TV/segmentation method is even superior to direct segmentation after subtraction of the median (Steps 1, 3 and 4 of Algorithm 1).

As the patterns within the Orangutan retina closely resemble the organization within the human one, similar results are to be expected for human samples.

Let us briefly compare the proposed method with other approaches pursued in the literature. In [8], the authors perform the image processing steps by use of a commercial software package which, unfortunately, comes as a “black box” without documentation of the internally utilized algorithms. In [9,10,20,21], after certain presmoothing/denoising steps, watershed segmentation is employed. Additionally, before segmenting, in [20] an illumination correction is performed while the authors in [10] interpose a contrast enhancement step. A common feature of all approaches is the necessity to select a number of parameters, including the (expected or minimal) feature size, by the experimenter.

Although preprocessing steps, particularly denoising or smoothing of the raw image data, are crucial for the quality of the results of subsequent segmentation, they have not been thoroughly documented in the cited references (if at all), and their dependence on additional, manually tuned parameters remains unclear. Moreover, any denoising method generates artifacts, thus modifying fine structures within the images in a specific way. In contrast to this situation, the preprocessing steps involved in our method (median filtering and TV denoising) are traceable and reproducible, including the manual setting of the single parameter *m*. For the TV denoising method, the characteristic artifacts are well-investigated (see e. g. the discussion in [16], pp. 519 ff.). In fact, this method has been deliberately selected in order to take advantage of its well-known “cartooning” effect. As a further difference, we segment by intensity thresholding instead of watershedding. The latter approach is well suited for the analysis of large, clumpy cell aggregates while our method is better suited for the detection of single cells to be differentiated from a more or less blotchy background, which may contain additional structures like vessels or different cell types. The segmentation depends on no more than two further parameters *c* and *f*, which have to be selected on the base of the observed contrast as well as of a reasonable guess of the feature size. A possible improvement could be the introduction of an additional upper bound for the size of the recognized features, thus reducing and possibly underestimating the number of cells since larger aggregates formed of merged cell images will then be excluded.

The reliability of our method, when evaluated by the mean relative error of automatic counting in relation to manual counts (5.9% as documented above), fits well within the range of errors documented in the cited references: [9], p. 1969, Figure one: ≤ 10% in 11 of 23 cases; [10], p. 641, Figure two: ≤ 5% in 31 of 40 cases; [20], p. R100.7, Figure two(A): 6% and 17%; [21], p. 589, Figure three: ≤ 10% in roughly half of the cases.

Due to its traceability, the TV denoising method offers the further advantage of easy reimplementation. On the one hand, this is possible with respect to the use of non-proprietary software, on the other hand, the method may be carried over (after optimization of the code as necessary) to the analysis of three- or multidimensional data stacks. Concerning the runtime behaviour, the analysis of an 1024 ×1024-pixel image takes typically less than 40 sec (on a Mini-PC equipped with four processors Intel(R)Core(TM)i3 CPU M380 @ 2.53 GHz) where approximately half of the time is consumed by the median filtering procedure. No particular attempts for tuning have been made.

**Figure 4:**
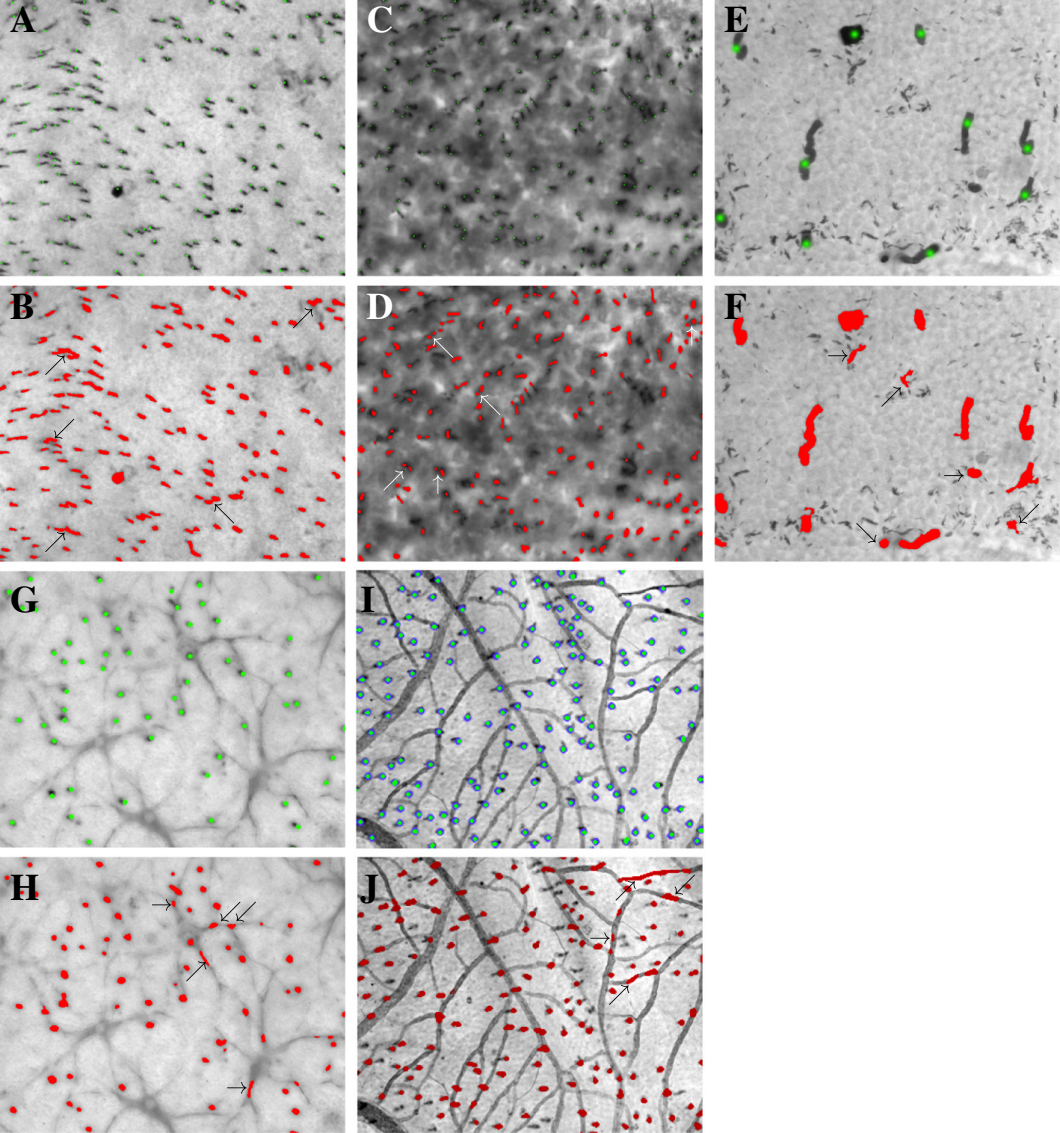
**Typical errors in automatic counting.** Sample clips with manually counted photoreceptors (tagged with green or blue dots, respectively) (A,C,E,G,I) will be compared with automatic counts obtained with Algorithm 1 (counted features dyed in red) (B,D,F,H,J). In the latter, the arrows point to occasional artifactual errors. **(A,B)** Lynx, No. 8: Adjacent cells have been merged and counted as a single one. **(C,D)** Pangolin, No. 12: Single cells are counted twice. **(E,F)** Agouti, No. 16: Background features (clustered melanin granules) and outer segment fragments are counted as cells. **(G,H)** Jaguar, No. 11: Parts of horizontal cells in the background are counted as S-cones. Note that, however, S-cones and horizontal cells are generally well differentiated, and even S-cones positioned directly above a horizontal cell are correctly recognized (middle left). **(I,J)** Orangutan, No. 2: Parts of vessels are counted as S-cones. In general, however, S-cones and vessels are well differentiated, and even S-cones positioned directly above a vessel are correctly recognized (left and middle part of the clip).

The limitations of the proposed method are exemplified in Figure 4. It shows typical errors within the automatic counting, which would be avoided by a human examiner. Adjacent cells are merged and counted as a single feature (Figure 4A and B). Another typical error occurs when a single photoreceptor cell does not lie exactly in the image plane. The resulting blurred image, may be “broken” into two or more features, resulting in double or multiple counts (Figure 4C and D). Also, cell debris and background spots may not be recognized as such (Figure 4E and F). Consequently, for heavily contaminated samples such as Nos. 16 and 18, a reduction of the quality of the automatic count is to be expected (in Table 2, No. 16 is the outlier). If background structures such as horizontal cells or vessels are present in the samples, it may happen that parts of them will be counted for cells as well (Figure 4G–J). Normally, however, the TV denoising method is well able to differentiate horizontal cells or vessels and even to recognize target cells, which are positioned immediately above a horizontal cell or a vessel. This is exemplarily shown in Figure 4G–J as well.

## Conclusions

In conclusion, the presented approach provides a reproducible method for the segmentation of labeled cellular objects, in particular retinal photoreceptors from grey scale micrographs. Relatively simple outlines and little overlap between the targets in the projection plane are clearly important preconditions for the current implementation, but within these limitations the procedure is shown to deliver robust results well comparable to those of manual counts by experienced observers. The method is well documented and can be transferred to other tasks including those with more automatization for both image acquisition and further refinements such as from 2D- or 3D-object recognition criteria.

## Availability and requirements

The software, to which applies the GNU General Public License v.2, is stored at the location http://www.meduniwien.ac.at/counttool/ within the archive cacount_tool_2013_02_08.zip. Its execution requires a current version of MATLAB (e.g., v. 7.14.0.739 (R2012a) and higher) together with the MATLAB Image Processing Toolbox (e.g., v. 8.0 (R2012a) and higher). Within this environment, the tool runs platform-independently. The code has been written in MATLAB; its listing will be provided as Additional file 1. To use the tool, deflate the zip-archive, include its location as well as location of the image data into the MATLAB path and type the command main. Further details on the usage can be found in the accompanying online documentation.

## Appendix

### Rudin-Osher-Fatemi TV denoising: mathematical background

The Rudin-Osher-Fatemi TV denoising procedure fits into a framework where greyscale images will be modeled by “continuous” rather than by “discrete” mathematical objects. More precisely, a greyscale image will be identified with a function x(s):Ω→R, which is at least bounded and measurable. The commonly used model for capturing an original scene is the equation

(7) I(s)=S(x(s))+N(s)

where x(s):Ω→R is an “ideal” image of the scene,  is an operator encoding the known systematical errors of the imaging device and N(s) is a noise term, cf. [22], pp. 60 ff., and [23], pp. 7 ff. Due to the presence of N(s), the error in the formal solution of (7),

(8) $$x(s)\;=\;S^{-1}\left(\;I(s)\;\right)-S^{-1}\left(\;N(s)\;\right)\;,$$

cannot be controlled by the possible deviations within the captured data *I*(*s*) alone. In mathematical terms, the reconstruction of the “denoised” or “smoothed” image *x*(*s*) via (8) thus represents an “ill-posed problem”, which needs for regularization. In large parts of the literature, consequently, image denoising is performed by minimizing functionals of the type

(9) $$F(x)\;=\;\int_{\Omega }{\left(\;I(s)-S\left(\;x(s)\;\right)\;\right)}^{2}\;\text{ds}+\frac{\alpha }{2}\cdot R\left(\;\nabla x\;\right)$$

over suitable function spaces, e. g. spaces of Sobolev functions or functions of bounded variation. The first member within *F*, the data fidelity term, aims for a least-square approximation of the captured data *I*(*s*) while the second one, the so-called regularization term, has been purposely introduced in order to ensure existence as well as uniqueness of a minimizing solution *x*(*s*). The influence of the second term is weighted by a number *α* > 0, which is called the regularization parameter. Note that the regularization term depends on the gradient ∇*x*(*s*), thus favorizing a certain edge structure within the minimizing solution *x*(*s*). A rigorous mathematical development of this idea relies on a closer analysis of the Euler-Lagrange equation, which must be satisfied as a second-order PDE by the minimizers of (9) as a necessary condition, cf. [22], pp. 64–66.

In the Rudin-Osher-Fatemi TV denoising problem, the operator  within the data fidelity term is the identity S(x(s))=x(s). The regularization term, which favors a minimizing solution *x*(*s*) with a fairly accentuated edge structure, is taken as $R\left(\;\nabla x\;\right)\;=\;|\;x\;{|}_{\text{TV}}$ with

(10) $$\begin{array}{lll} & |\;x\;{|}_{\text{TV}}\;=\;\text{sup}\;\left\{\;\int_{\Omega }x(s)\;\left(\;\frac{\partial \psi_{1}}{\partial s_{1}}(s)+\frac{\partial \psi_{2}}{\partial s_{2}}(s)\;\right)\;\right)\; & \;\\ & \;\;\;\left(\times \text{ds}\;\;\left|\right)\;\;\psi_{1}(s)\;,\;\psi_{2}(s)\;:\;\;\Omega \to R\;\;\;\text{are}\right)\; & \;\\ & \;\text{continuously differentiable test functions,}\; & \;\\ & \;\text{taking zero boundary values and}\;\\ & \;\text{satisfying}\;\psi_{1}{\left(s\right)}^{2}\;+\;\psi_{2}{\left(s\right)}^{2}\leq 1\;\text{everywhere on}\;\Omega \;\})\;,\; & \; \end{array}$$

and the minimization is performed over all functions of bounded variation on *Ω* (for more details, cf. [24], pp. 355 ff., and [25], pp. 175 ff.). Loosely speaking, | *x* |_*T* *V*_ is a measure for the oscillation of the function *x*(*s*) and depends on the (generalized) derivatives of *x*(*s*) through a duality expression. A closer mathematical analysis reveals that the solution *x*(*s*) of the Rudin-Osher-Fatemi TV denoising problem largely preserves the edge structure of the input data *I*(*s*). After a suitable discretization, the particular structure of | *x* |_*T* *V*_ allows for the application of highly efficient primal-dual numerical solvers like the one utilized in our present study, cf. again [25], pp. 179 ff.

## Competing interests

The authors declare that they have no competing interests.

## Authors’ contributions

PA and CS provided the retinal samples and collected the image data. CS performed the manual counts. KB suggested the application of the TV procedure and programmed the tool. MW performed the numerical experiments and drafted the manuscript. All authors read and approved the final manuscript.

## Pre-publication history

The pre-publication history for this paper can be accessed here:

http://www.biomedcentral.com/1471-2415/13/59/prepub

## Supplementary Material

Additional file 1 Appendix: Documentation of the counting tool. Click here for file
