# Supplementary material for: Computer-assisted counting of retinal cells by automatic segmentation after TV denoising
Source: BMC Ophthalmol. 2013 Oct 20;13:59. doi: 10.1186/1471-2415-13-59 (PMC3835550; doi:10.1186/1471-2415-13-59)
Supplement: Additional file 1 — Appendix: Documentation of the counting tool. [file 1471-2415-13-59-S1.pdf]

Computer-assisted counting of retinal cells by automatic segmentation after TV denoising

Kristian Bredies, Marcus Wagner, Christian Schubert and Peter Ahnelt

Appendix: Documentation of the counting tool.

a) Files contained within the package.

```
CellCount
CellCount.bat
main.m
mesg.m
process.m
report.m
about_ui.fig
main_ui.fig
rep_gen_ui.fig
seg_conf_ui.fig
README
gpl-2.0-standalone.html
doc/short_instructions.html
doc/short_instructions_html_486c2b0b.jpg
doc/short_instructions_html_4e2f1fe2.jpg
doc/short_instructions_html_m1005195.jpg
doc/short_instructions_html_m25a5059e.jpg
doc/short_instructions_html_m2964736c.jpg
doc/short_instructions_html_m5f21bf5e.jpg
```

b) Listing of `main.m`.

% main.m – main graphical user interface for cell counting tool

% Copyright (C) 2011–2012 Kristian Bredies (kristian.bredies@uni-graz.at)

% This program is free software; you can redistribute it and/or modify it under the terms of the GNU General Public % License as published by the Free Software Foundation; either version 2, or (at your option) any later version. % This program is distributed in the hope that it will be useful, but WITHOUT ANY WARRANTY; without % even the implied warranty of MERCHANTABILITY or FITNESS FOR A PARTICULAR PURPOSE. See the % GNU General Public License for more details. You should have received a copy of the GNU General Public License % along with this program; if not, write to the Free Software Foundation, Inc., 51 Franklin Street - Fifth Floor, % Boston, MA 02110-1301, USA.

```
function main(varargin)

  %%%%%% initialize

  param.action = 'default'; param.files = {}; param.debug = 1; param = process(param);

  %%%%%% load GUI

  hwnd = openfig('main_ui.fig'); figure(hwnd); gui = get_gui_handles();
  set(gui.status, 'String', 'Ready.'); initialize_callbacks(); update_gui_elements();
```

```
  %%%%%% check for call with 'quit'

  quit_on_close = 0;
    if (nargin == 1)
      if (strcmp(varargin{1}, 'quit'))
        quit_on_close = 1;
      end
    end

%%%%%% callback functions

%%% segmentation
function do_counting(obj, event)
  data = guidata(gcbo);
  if (isfield(data, 'exe'))
    % give signal to terminate
    mesg(1, 'Sending termination request...'); data.abort = 1; guidata(gcbo, data);
  else
    % perform action
    param.action = 'do'; oldstr = get(gui.seg.do, 'String'); set(gui.seg.do, 'String', 'Abort');
    data.exe = 1; guidata(gcbo, data);
    try
      param_discard = process(param);
    catch ME
      message = regexprep(ME.message, '<(.*?)>', ' ');
      mesg(1, sprintf('Exception (%s)', ME.identifier)); mesg(1, message); stack = 'Stack: ';
      for i = 1:length(ME.stack)
        stack = strcat(stack, sprintf('%d@%s; ', ME.stack(i).line, ME.stack(i).name));
      end
      mesg(1, stack);
    end
    data = guidata(gcbo); data = rmfield(data,'exe'); guidata(gcbo, data);
    set(gui.seg.do, 'String', oldstr);
  end
end

function counting_conf(obj, event)
  param.action = 'configure'; param = process(param);
end

%%% report
function generate_report(obj, event)
  param.action = 'generate'; param = report(param);
end

%%% menu routines
function menu_load(obj, event)
  [name, path] = uigetfile('*.mat','Load job');
  if (ischar(name))
    contents = load([path name], 'param'); param = contents.param;
    % add path of the job file if necessary
    if (isfield(param, 'files'))
      for i = 1:length(param.files)
        [pathstr, name, ext] = fileparts(param.files{i});
        if (isempty(pathstr))
          param.files{i} = [path name ext];
```

```
        end
      end
    end
    update_gui_elements();
  end
end

function menu_save(obj, event)
  [name, path] = uiputfile('*.mat', 'Save job as');
  if (ischar(name))
    save([path name], 'param');
  end
end

function menu_quit(obj, event)
  if (strcmp(questdlg('Really quit?'), 'Yes'))
    delete(gcbf);
    if (quit_on_close)
      exit;
    end
  end
end

function menu_about(obj, event)
  hdlg = openfig('about_ui.fig'); waitfor(hdlg);
end

function menu_help(obj, event)
  web('doc/short_instructions.html');
end

%%% file selection stuff
function files_add(obj, event)
  [name, path] = uigetfile({'*.bmp;*.gif;*.jpg;*.jpeg;*.png;*.tif;*.tiff; ...
  *.mat', 'All image formats'; '*.bmp', 'Windows bitmap (*.bmp)'; ...
  '*.gif', 'Graphics interchange format (*.gif)'; ...
  '*.jpg;*.jpeg', 'Joint photographic experts group (*.jpg, *.jpeg)'; ...
  '*.png', 'Portable network graphics (*.png)'; ...
  '*.tif;*.tiff', 'Tagged image file format (*.tif, *.tiff)'; ...
  '*.mat', 'AMIRA Matlab export format (*.mat)'}, 'Select image files', 'Multiselect', 'on');
  if (ischar(name) || iscell(name))
    % get index to insert
    firstindex = get(gui.files, 'Value');
    if (isempty(firstindex))
      firstindex = 1;
    else
      firstindex = min(firstindex) + 1;
    end
    % deal with inconsistent uigetfile output
    if (iscell(name))
      for i = 1:length(name)
        name{i} = [path name{i}];
      end
    else
      name = [path name];
    end
```

```
    param.files = [param.files{1:firstindex-1} name param.files{firstindex:end}];
    selected = firstindex:(firstindex+length(name)-1); update_file_box(selected);
  end
end

function files_del(obj, event)
  sel = get(gui.files, 'Value');
  if (~isempty(sel))
    mask = ~ismember(1:length(param.files), sel); param.files = param.files(mask);
    update_file_box([]);
  end
end

function files_move_up(obj, event)
  sel = sort(get(gui.files, 'Value')); N = length(param.files);
  % insert dummy (0)
  perm = 0:N;
  % permute according to selection
  for i = 1:length(sel)
    j = sel(i);
    if (j <= N)
      swap = perm(j+1); perm(j+1) = perm(j); perm(j) = swap;
    end
  end
  % remove dummy
  perm = perm(perm ~= 0);
  % compute inverse permutation
  invperm = zeros(N,1); invperm(perm) = 1:N;
  % apply permutation und update
  files = cell(N,1);
  for i = 1:N
    files{i} = param.files{perm(i)};
  end
  param.files = files; newsel = invperm(sel); update_file_box(newsel);
end

function files_move_down(obj, event)
  sel = sort(get(gui.files,'Value'),2,'descend'); N = length(param.files);
  % insert dummy (0)
  perm = [1:N 0];
  % permute according to selection
  for i = 1:length(sel)
    j = sel(i);
    if (j <= N)
      swap = perm(j+1); perm(j+1) = perm(j); perm(j) = swap;
    end
  end
  % remove dummy
  perm = perm(perm ~= 0);
  % compute inverse permutation
  invperm = zeros(N,1); invperm(perm) = 1:N;
  % apply permutation und update
  files = cell(N,1);
  for i = 1:N
    files{i} = param.files{perm(i)};
```

```
  end
  param.files = files; newsel = invperm(sel); update_file_box(newsel);
end

%%% replacement rule stuff
function file_pat_changed(obj, event)
  str = get(gui.file.fluor_pat, 'String'); param.seg.post_fluor_pat = str;
end

function file_rep_changed(obj, event)
  str = get(gui.file.fluor_rep, 'String'); param.seg.post_fluor_rep = str;
end

function gen_img_changed(obj, event)
  val = get(gui.file.gen_img, 'Value'); param.seg.post_gen_img = val;
end

%%%%%% gui stuff

function update_file_box(sel)
  str = cell(length(param.files), 1);
  for i=1:length(param.files)
    [path,name,ext] = fileparts(param.files{i}); str{i} = [name ext];
  end
  set(gui.files, 'ListboxTop', min(max(1,length(param.files)), ...
  max(1, get(gui.files, 'ListboxTop'))));
  set(gui.files, 'String', str); set(gui.files, 'Value', sel);
end

function update_gui_elements()
  % sets the adjustable elements according to param
  update_file_box([]); set(gui.files, 'Value', []);
  set(gui.file.fluor_pat, 'String', param.seg.post_fluor_pat);
  set(gui.file.fluor_rep, 'String', param.seg.post_fluor_rep);
  set(gui.file.gen_img, 'Value', param.seg.post_gen_img);
end

function initialize_callbacks()
  % set all callback functions
  set(hwnd, 'CloseRequestFcn', @menu_quit);
  % menu
  set(gui.menu.load, 'Callback', @menu_load); set(gui.menu.save, 'Callback', @menu_save);
  set(gui.menu.quit, 'Callback', @menu_quit); set(gui.menu.about, 'Callback', @menu_about);
  set(gui.menu.help, 'Callback', @menu_help);
  % files selection
  set(gui.file.add, 'Callback', @files_add); set(gui.file.del, 'Callback', @files_del);
  set(gui.file.up, 'Callback', @files_move_up); et(gui.file.down, 'Callback', @files_move_down);
  % replacement rule
  set(gui.file.fluor_pat, 'Callback', @file_pat_changed);
  set(gui.file.fluor_rep, 'Callback', @file_rep_changed);
  set(gui.file.gen_img, 'Callback', @gen_img_changed);
  % segmentation buttons
  set(gui.seg.do, 'Callback', @do_counting); set(gui.seg.conf, 'Callback', @counting_conf);
  % report buttons
  set(gui.report.do, 'Callback', @generate_report);
end

function gui = get_gui_handles()
```

```
  % output elements
  gui.axes = findobj(hwnd, 'Tag', 'outputwindow');
  gui.status = findobj(hwnd, 'Tag', 'outputtext');
  % menu
  gui.menu.load = findobj(hwnd, 'Tag', 'load'); gui.menu.save = findobj(hwnd, 'Tag', 'save');
  gui.menu.quit = findobj(hwnd, 'Tag', 'quit');
  gui.menu.help = findobj(hwnd, 'Tag', 'instructions');
  gui.menu.about = findobj(hwnd, 'Tag', 'about');
  % file dialog
  gui.files = findobj(hwnd, 'Tag', 'files'); gui.file.add = findobj(hwnd, 'Tag', 'files_add');
  gui.file.del = findobj(hwnd, 'Tag', 'files_remove');
  gui.file.up = findobj(hwnd, 'Tag', 'files_move_up');
  gui.file.down = findobj(hwnd, 'Tag', 'files_move_down');
  gui.file.fluor_pat = findobj(hwnd, 'Tag', 'fluor_pat');
  gui.file.fluor_rep = findobj(hwnd, 'Tag', 'fluor_rep');
  gui.file.gen_img = findobj(hwnd, 'Tag', 'generate_seg_image');
  % segmentation dialog
  gui.seg.do = findobj(hwnd, 'Tag', 'counting_do');
  gui.seg.conf = findobj(hwnd, 'Tag', 'counting_configure');
  % report dialog
  gui.report.do = findobj(hwnd, 'Tag', 'report_do');
  gui.report.expl = findobj(hwnd, 'Tag', 'report_explore');
end
end
```

c) Listing of `mesg.m`.

```
% mesg.m - displays output messages

% Copyright (C) 2008–2011 Kristian Bredies (kristian.bredies@uni-graz.at), 2010–2011 Florian Leitner (florian.leitner
% @student.tugraz.at)

% This program is free software; you can redistribute it and/or modify it under the terms of the GNU General Public
% License as published by the Free Software Foundation; either version 2, or (at your option) any later version.
% This program is distributed in the hope that it will be useful, but WITHOUT ANY WARRANTY; without
% even the implied warranty of MERCHANTABILITY or FITNESS FOR A PARTICULAR PURPOSE. See the
% GNU General Public License for more details. You should have received a copy of the GNU General Public License
% along with this program; if not, write to the Free Software Foundation, Inc., 51 Franklin Street - Fifth Floor,
% Boston, MA 02110-1301, USA.

function mesg(debug, str)

  % do not print empty string
  if (isempty(str))
    return;
  end
  if (debug)
    if (isempty(gcbf))
      % remove carriage return if necessary
      if (str(1) == 13)
        str = str(2:end);
      end
      disp(str);
    else
      hwnd = gcbf(); status = findobj(hwnd, 'Tag', 'outputtext'); text = get(status, 'String');
      % if first character is carriage return, then replace line
```

```
      if (str(1) == 13)
        text{end} = str;
      else
        if (length(text) >= 500)
          text = text(2:500);
        end
        text = cat(1, text, {str});
      end
      set(status, 'String', text); set(status, 'Value', length(text)); drawnow;
      % check for terminate request
      data = guidata(gcbo);
      if (isfield(data, 'abort'))
        data = rmfield(data, 'abort'); guidata(gcbo, data);
        throw(MException('User:Abort', 'User terminated execution'));
      end
    end
  end
end
```

d) Listing of `process.m`.

```
% process.m – cell counting module

% Copyright (C) 2011–2012 Kristian Bredies (kristian.bredies@uni-graz.at)

% This program is free software; you can redistribute it and/or modify it under the terms of the GNU General Public
% License as published by the Free Software Foundation; either version 2, or (at your option) any later version.
% This program is distributed in the hope that it will be useful, but WITHOUT ANY WARRANTY; without
% even the implied warranty of MERCHANTABILITY or FITNESS FOR A PARTICULAR PURPOSE. See the
% GNU General Public License for more details. You should have received a copy of the GNU General Public License
% along with this program; if not, write to the Free Software Foundation, Inc., 51 Franklin Street - Fifth Floor,
% Boston, MA 02110-1301, USA.

% – cell counting configuration dialog
% – batch processing

% param: parameter structure – fields:
% action – 'default' returns a default configuration, 'configure' invokes configuration dialog, 'do' performs cell counting
% files – list of images to process
% seg – counting parameter structure
% debug – if set, debug output will be produced

function param = process(param)
  % check for action
  if (~isfield(param, 'action'))
    mesg(1, 'Could not determine what to do.'); return;
  end
  isdebug = isfield(param, 'debug'); action = 0;
  if (strcmp(param.action, 'default'))
    % create default configuration
    action = 1; mesg(isdebug, 'Creating default configuration.');
    param = configure_default(param);
  end
  if (strcmp(param.action, 'configure'))
    % configuration
    action = 1; mesg(isdebug, 'Invoking configuration dialog.');
```

```
  param = configure_segmentation(param);
end
if (strcmp(param.action, 'do'))
  % batch processing
  action = 1;
  % check for file list
  if (~isfield(param, 'files'))
    mesg(1, 'No files to process specified.'); return;
  end
  numfiles = length(param.files);
  if (numfiles == 0)
    mesg(1, 'No files to process specified.'); return;
  end
  % process in a for loop
  for fnum = 1:numfiles
    curfile = param.files{fnum}; [pathstr, name, ext] = fileparts(curfile);
    resfile = fullfile(pathstr, [name '_res.mat']);
    if (isfield(param, 'seg'))
      if (isfield(param.seg, 'post_fluor_pat') && (isfield(param.seg, 'post_fluor_rep')))
        if (strfind(param.seg.post_fluor_pat,'.'))
          % replace name and extension
          segfile = fullfile(pathstr, strrep([name ext], ...
          param.seg.post_fluor_pat, param.seg.post_fluor_rep));
        else
          % replace name only
          segfile = fullfile(pathstr, [strrep(name, param.seg.post_fluor_pat, ...
          param.seg.post_fluor_rep) ext]);
        end
      else
        segfile = curfile;
      end
    end
    mesg(1, sprintf('[%d/%d] Processing %s...', fnum, numfiles, curfile));
    mesg(1, '-----------------------------------------------------------------');
    mesg(1, 'Parameters:');
    mesg(1, '-----------------------------------------------------------------');
    mesg(1, 'median filter size (m) / segmentation threshold (c) / feature size (f):');
    mesg(1, sprintf('*** m / c / f = %d / %f / %d ***', param.seg.pre_medfilt_len, ...
    param.seg.pre_seg_thres, param.seg.pre_feat_size));
    if (param.seg.pre_tv)
      mesg(1, 'total variation regularization (alpha) / iteration count (N):');
      mesg(1, sprintf('*** alpha / N = %f / %d ***', param.seg.pre_tv_reg, ...
      param.seg.pre_tv_iter));
    end
    mesg(1, '-----------------------------------------------------------------');
    % handle the case of equal filenames
    if ((param.seg.post_gen_img) && (strcmp(segfile, curfile)))
      segfile = fullfile(pathstr, [name '_seg' ext]);
      mesg(1, sprintf(['Warning: Input file and segmented file are identical.'...
      'Outputting to %s.'], segfile));
    end
    % processing
    if (param.seg.process)
```

```
        % load image and convert
        if (exist(curfile, 'file'))
          image = loadimage(param, curfile);
          mesg(isdebug, sprintf('Image file ''%s'' loaded and converted.', curfile));
          param = process_image(param, image, segfile, resfile);
        else
          mesg(1, sprintf('Image file ''%s'' does not exist. Skipped.', curfile));
        end
      end
      mesg(isdebug, sprintf('[%d/%d] Processing of ''%s'' complete.', fnum, numfiles, curfile));
    end
  end
  if (action == 0)
    mesg(1, sprintf('Action ''%s'' not implemented.', param.action));
  end
end

% create default configuration
function param = configure_default(param)
  % general parameters
  seg.crop_left = 3; seg.crop_right = 3; seg.crop_top = 3; seg.crop_bottom = 3;
  seg.pre_scaleimage = 0; seg.pre_scaleimage_width = 1024; seg.pre_scaleimage_height = 1024;
  % processing parameters
  seg.process = 1; seg.pre_medfilt_len = 30;
  % tv regularization
  seg.pre_tv = 1; seg.pre_tv_reg = 0.05; seg.pre_tv_iter = 50;
  % segmentation parameter
  seg.pre_seg_thres = 2.5; seg.pre_feat_size = 5;
  % file name replace defaults
  seg.post_fluor_pat = '.tif'; seg.post_fluor_rep = '_seg.tif'; seg.post_gen_img = 1;
  param.seg = seg;
end

function image = loadimage(param, fname)
  % loads an image, crops and converts it
  [pathstr, name, ext] = fileparts(fname);
  % treat AMIRA Matlab files differently
  if (strcmp(ext, '.mat'))
    vars = load(fname); fieldn = fieldnames(vars);
    if (length(fieldn) >= 1)
      image = vars.(fieldn{1});
      % transpose since AMIRA swaps dim 1 and 2
      image = permute(image, [2 1 3]);
    else
      % check for pathological case
      mesg(1, sprintf('File ''%s'' does not contain any data.', fname)); image = 0;
    end
  else
    image = imread(fname);
  end
  % get cropping parameters
  if (isfield(param, 'seg'))
    if (isfield(param.seg, 'crop_left'))
      cl = param.seg.crop_left;
```

```
    else
      cl = 0;
    end
    if (isfield(param.seg, 'crop_right'))
      cr = param.seg.crop_right;
    else
      cr = 0;
    end
    if (isfield(param.seg, 'crop_top'))
      ct = param.seg.crop_top;
    else
      ct = 0;
    end
    if (isfield(param.seg, 'crop_bottom'))
      cb = param.seg.crop_bottom;
    else
      cb = 0;
    end
  else
    cl = 0; cr = 0; ct = 0; cb = 0;
  end
  % crop image
  left = cl + 1; right = size(image,2) - cr;
  if (right < 1)
    right = 1;
  end
  if (left > right)
    left = right;
  end
  top = ct + 1; bottom = size(image,1) - cb;
  if (bottom < 1)
    bottom = 1;
  end
  if (top > bottom)
    top = bottom;
  end
  image = image(top:bottom,left:right,:); image = double(image);
  % convert to graylevel in case of 2d images
  if (~strcmp(ext,'.mat'))
    % to black and white
    if (size(image,3) == 3)
      image = 0.3*image(:,:,1) + 0.59*image(:,:,2) + 0.11*image(:,:,3);
    end
    % take first channel if not RGB
    if (size(image, 3) > 1)
      image = image(:,:,1);
    end
  end
end

% configuration dialog
function param = configure_segmentation(param)
  param_old = param; hdlg = openfig('seg_conf_ui.fig');
  gui = get_gui_handles(); initialize_bounds(); initialize_values(); initialize_callbacks();
```

```
save_settings = 0; waitfor(hdlg);
if (~save_settings)
  param = param_old;
end

% callbacks
function okay_button(obj, event)
  save_values(); save_settings = 1; close(gcbf);
end

function cancel_button(obj, event)
  close(gcbf);
end

function reset_button(obj, event)
  param_new = configure_default(param);
  % hack for main gui parameters
  param_new.seg.post_fluor_pat = param.seg.post_fluor_pat;
  param_new.seg.post_fluor_rep = param.seg.post_fluor_rep;
  param = param_new; initialize_values();
end

function check_bounds(obj, event)
  % checks bound for numeric values
  bounds = get(gcbo, 'UserData'); num = str2double(get(gcbo, 'String'));
  num = min(bounds(2),max(bounds(1),num)); set(gcbo, 'String', num2str(num));
end

% initalization and save routines
function initialize_callbacks()
  % pushbutton callbacks
  set(gui.okay, 'Callback', @okay_button); set(gui.reset, 'Callback', @reset_button);
  set(gui.cancel, 'Callback', @cancel_button);
% bounds check for all numeric values
  set(gui.crop_left, 'Callback', @check_bounds); set(gui.crop_right, 'Callback', @check_bounds);
  set(gui.crop_top, 'Callback', @check_bounds); set(gui.crop_bottom, 'Callback', @check_bounds);
  set(gui.pre_scaleimage_width, 'Callback', @check_bounds);
  set(gui.pre_scaleimage_height, 'Callback', @check_bounds);
  set(gui.pre_medfilt_len, 'Callback', @check_bounds);
  set(gui.pre_tv_reg, 'Callback', @check_bounds);
  set(gui.pre_tv_iter, 'Callback', @check_bounds);
  set(gui.pre_seg_thres, 'Callback', @check_bounds);
  set(gui.pre_feat_size, 'Callback', @check_bounds);
end

function initialize_values()
  set(gui.crop_left, 'String', num2str(param.seg.crop_left));
  set(gui.crop_right, 'String', num2str(param.seg.crop_right));
  set(gui.crop_top, 'String', num2str(param.seg.crop_top));
  set(gui.crop_bottom, 'String', num2str(param.seg.crop_bottom));
  set(gui.process, 'Value', param.seg.process);
  set(gui.pre_scaleimage, 'Value', param.seg.pre_scaleimage);
  set(gui.pre_scaleimage_width, 'String', num2str(param.seg.pre_scaleimage_width));
  set(gui.pre_scaleimage_height, 'String', num2str(param.seg.pre_scaleimage_height));
  set(gui.pre_medfilt_len, 'String', num2str(param.seg.pre_medfilt_len));
  set(gui.pre_tv, 'Value', param.seg.pre_tv);
```

```
    set(gui.pre_tv_reg, 'String', num2str(param.seg.pre_tv_reg));
    set(gui.pre_tv_iter, 'String', num2str(param.seg.pre_tv_iter));
    set(gui.pre_seg_thres, 'String', num2str(param.seg.pre_seg_thres));
    set(gui.pre_feat_size, 'String', num2str(param.seg.pre_feat_size)); end

  function save_values()
    param.seg.crop_left = str2double(get(gui.crop_left, 'String'));
    param.seg.crop_right = str2double(get(gui.crop_right, 'String'));
    param.seg.crop_top = str2double(get(gui.crop_top, 'String'));
    param.seg.crop_bottom = str2double(get(gui.crop_bottom, 'String'));
    param.seg.process = get(gui.process, 'Value');
    param.seg.pre_scaleimage = get(gui.pre_scaleimage, 'Value');
    param.seg.pre_scaleimage_width = str2double(get(gui.pre_scaleimage_width, 'String'));
    param.seg.pre_scaleimage_height = str2double(get(gui.pre_scaleimage_height, 'String'));
    param.seg.pre_medfilt_len = str2double(get(gui.pre_medfilt_len, 'String'));
    param.seg.pre_tv = get(gui.pre_tv, 'Value');
    param.seg.pre_tv_reg = str2double(get(gui.pre_tv_reg, 'String'));
    param.seg.pre_tv_iter = str2double(get(gui.pre_tv_iter, 'String'));
    param.seg.pre_seg_thres = str2double(get(gui.pre_seg_thres, 'String'));
    param.seg.pre_feat_size = str2double(get(gui.pre_feat_size, 'String'));
  end

  function initialize_bounds()
    set_bounds(gui.crop_left, 0, 10000); set_bounds(gui.crop_right, 0, 10000);
    set_bounds(gui.crop_top, 0, 10000); set_bounds(gui.crop_bottom, 0, 10000);
    set_bounds(gui.pre_scaleimage_width, 16, 4096);
    set_bounds(gui.pre_scaleimage_height, 16, 4096);
    set_bounds(gui.pre_medfilt_len, 1, 10000); set_bounds(gui.pre_tv_reg, 0, 1000);
    set_bounds(gui.pre_tv_iter, 0, 1000000); set_bounds(gui.pre_seg_thres, 0, 1000);
    set_bounds(gui.pre_feat_size, 0, 10000);
  end

  function set_bounds(handle, low, high)
    bounds = [low high]; set(handle, 'UserData', bounds);
  end

  function gui = get_gui_handles()
    gui.okay = findobj(hdlg, 'Tag', 'okay'); gui.reset = findobj(hdlg, 'Tag', 'reset');
    gui.cancel = findobj(hdlg, 'Tag', 'cancel'); gui.crop_left = findobj(hdlg, 'Tag', 'crop_left');
    gui.crop_right = findobj(hdlg, 'Tag', 'crop_right');
    gui.crop_top = findobj(hdlg, 'Tag', 'crop_top');
    gui.crop_bottom = findobj(hdlg, 'Tag', 'crop_bottom');
    gui.process = findobj(hdlg, 'Tag', 'process');
    gui.pre_scaleimage = findobj(hdlg, 'Tag', 'pre_scaleimage');
    gui.pre_scaleimage_width = findobj(hdlg, 'Tag', 'pre_scaleimage_width');
    gui.pre_scaleimage_height = findobj(hdlg, 'Tag', 'pre_scaleimage_height');
    gui.pre_medfilt_len = findobj(hdlg, 'Tag', 'pre_medfilt_len');
    gui.pre_tv = findobj(hdlg, 'Tag', 'pre_tv');
    gui.pre_tv_reg = findobj(hdlg, 'Tag', 'pre_tv_reg');
    gui.pre_tv_iter = findobj(hdlg, 'Tag', 'pre_tv_iter');
    gui.pre_seg_thres = findobj(hdlg, 'Tag', 'pre_seg_thres');
    gui.pre_feat_size = findobj(hdlg, 'Tag', 'pre_feat_size');
  end
end

% processing part
```

```
function param = process_image(param, image, segfile, resfile)
  % normalize and scale
  if (param.seg.pre_scaleimage)
    image = imresize(image, [param.seg.pre_scaleimage_height param.seg.pre_scaleimage_width]);
  end
  if (max(image(:)) <= 255)
    im = double(image)/255;
  else
    im = double(image)/max(image(:));
  end
  imagesc(im); axis equal; colormap(gray(256)); drawnow;
  % do median filtering and subtract background
  mesg(1, 'Starting median filtering...');
  im2 = medfilt2(im, [param.seg.pre_medfilt_len param.seg.pre_medfilt_len], 'symmetric');
  im3 = im - im2; imagesc(im3); axis equal; colormap(gray(256)); drawnow;
  % do total variation smoothing
  if (param.seg.pre_tv)
    mesg(1, 'Total variation smoothing...');
    im4 = tgv1_l2_2D_pd(im3, param.seg.pre_tv_reg, param.seg.pre_tv_iter);
  else
    im4 = im3;
  end
  % do segmentation
  mesg(1, 'Starting segmentation...');
  m = mean(im4(:)); v = var(im4(:)); seg = (im4 < m - param.seg.pre_seg_thres*sqrt(v));
  % eliminate small features
  seg2 = bwlabel(seg); maxlabel = max(seg2(:));
  for i = 1:maxlabel
    if (sum(seg2(:) == i) < param.seg.pre_feat_size)
      seg2(seg2 == i) = 0;
    end
  end
  seg = seg2; found_cell_number = double(length(union(seg2(seg2 > 0),[])));
  [width_im4 height_im4] = size(im4);
  mean_density = double(100*found_cell_number/ ((width_im4 ...
  + 2*param.seg.pre_scaleimage_width)*(height_im4 + 2*param.seg.pre_scaleimage_height)));
  % save image
  if (param.seg.post_gen_img)
    mesg(1, sprintf('Writing labelled image %s...', segfile));
    res = (seg > 0); outline = own_dilate(res) - res; outline = (outline > 0);
    res_r = im; res_r(outline) = 0.5 + 0.5*res_r(outline);
    res_g = im; res_g(outline) = 0;
    res_b = im; res_b(outline) = 0;
    res = cat(3, res_r, res_g, res_b);
    imagesc(res); axis equal; colormap(gray(256)); drawnow; imwrite(res, segfile);
  end
  % save results
  mesg(1, sprintf('Found %d cells.', found_cell_number));
  mesg(1, sprintf('Mean density: %d cells per 10 x 10 px.', mean_density));
  save(resfile, 'seg');
end

function F = own_dilate(G)
  % hand-made 5-point dilation
```

```
  G1 = [G(2:end,:); G(end,:)]; G2 = [G(1,:); G(1:end-1,:)];
  G3 = [G(:,2:end) G(:,end)]; G4 = [G(:,1) G(:,1:end-1)];
  F = max(max(max(max(G,G1),G2),G3),G4);
end

function u = tgv1_l2_2D_pd(f, alpha0, maxits)
  check_it = 25; [M N] = size(f); xi = zeros(M,N,2); u = f;
  % multiindices of the spatial derivatives
  % derivatives
  % | uxx uxy |
  % | uyx uyy |
  % multiindices
  % | 1 3 |
  % | 3 2 |
  L = sqrt(8); tau_p = 1/L; tau_d = 1/L;
  for k = 0:maxits
    % PRIMAL UPDATE
    div = dxm(xi(:,:,1)) + dym(xi(:,:,2));
    % remember old u
    u_ = -u;
    % primal update
    u = (u + tau_p*div);
    % proximal operator
    u = (u + tau_p*f)./(1.0+tau_p);
    % over-relaxation
    u_ = u_ + 2.0*u;
    % DUAL UPDATE
    u_x = dxp(u_); u_y = dyp(u_);
    xi(:,:,1) = xi(:,:,1) + tau_d*u_x; xi(:,:,2) = xi(:,:,2) + tau_d*u_y;
    % REPROJECTION
    % compute minimizer of min_{xi} || xi-xi_old ||^2 s.t. || xi || <= alpha0
    % initialization
    denom = max(1.0, sqrt(xi(:,:,1).^2 + xi(:,:,2).^2)/alpha0);
     xi(:,:,1) = xi(:,:,1)./denom; xi(:,:,2) = xi(:,:,2)./denom;
    if mod(k,check_it) == 0
      imagesc(u); axis equal; colormap(gray(256)); drawnow;
    end
  end
end

function [dx] = dxm(u)
  M = size(u,1); dx = [u(:,1:end-1) zeros(M,1)] - [zeros(M,1) u(:,1:end-1)];
end

function [dx] = dxp(u)
  dx = [u(:,2:end) u(:,end)] - u;
end

function [dy] = dym(u)
  N = size(u,2); dy = [u(1:end-1,:);zeros(1,N)] - [zeros(1,N);u(1:end-1,:)];
end

function [dy] = dyp(u)
  dy = [u(2:end,:); u(end,:)] - u;
end
```

e) Listing of `report.m`.

% report.m – report generation module

% Copyright (C) 2011–2012 Kristian Bredies (kristian.bredies@uni-graz.at)

% This program is free software; you can redistribute it and/or modify it under the terms of the GNU General Public
% License as published by the Free Software Foundation; either version 2, or (at your option) any later version.
% This program is distributed in the hope that it will be useful, but WITHOUT ANY WARRANTY; without
% even the implied warranty of MERCHANTABILITY or FITNESS FOR A PARTICULAR PURPOSE. See the
% GNU General Public License for more details. You should have received a copy of the GNU General Public License
% along with this program; if not, write to the Free Software Foundation, Inc., 51 Franklin Street - Fifth Floor,
% Boston, MA 02110-1301, USA.

% – generates basic reports
% – provides a data exploration gui

% param: parameter structure – fields:
% action – 'generate' generates report after asking user, 'explore' invokes data exploration dialog
% files – list of images to process
% debug – if set, debug output will be produced

```
function param = report(param)
  % check for action
  if (~isfield(param, 'action'))
    mesg(1, 'Could not determine what to do.'); return;
  end
  isdebug = isfield(param, 'debug'); action = 0;
  if (strcmp(param.action, 'generate'))
    % configuration
    action = 1;
    % check for file list
    if (~isfield(param, 'files'))
      mesg(1, 'No files to process specified.'); return;
    end
    numfiles = length(param.files);
    if (numfiles == 0)
      mesg(1, 'No files to process specified.'); return;
    end
    % first ask for filename etc.
    mesg(isdebug, 'Invoking export dialog.'); [param, okay] = configure_report(param);
    if (okay)
      mesg(isdebug, 'Generating report...'); param = generate_report(param);
    else
      mesg(isdebug, 'Report generation canceled.');
    end
  end
  if (strcmp(param.action, 'explore'))
    % batch processing
    action = 1; mesg(isdebug, 'Invoking data exploration dialog.');
    mesg(isdebug, 'Sorry, not implemented yet.');
  end
  if (action == 0)
    mesg(1, sprintf('Action ''%s'' not implemented.', param.action));
  end
end
```

```
% report configuration
function [param, save_settings] = configure_report(param)
  param_old = param; hdlg = openfig('rep_gen_ui.fig');
  if (~isfield(param, 'report'))
    % initialize with default values
    param.report.outputfile = 'report.csv';
  end
  gui = get_gui_handles(); initialize_values(); initialize_callbacks();
  save_settings = 0; waitfor(hdlg);
  if (~save_settings)
    param = param_old;
  end

  % callbacks
  function okay_button(obj, event)
    save_values(); save_settings = 1; close(gcbf);
  end

  function cancel_button(obj, event)
    close(gcbf);
  end

  function filename_select(obj, event)
    [name, path] = uiputfile({'*.csv', 'Comma separated values (*.csv)'}, 'Save report as');
    if (ischar(name))
      param.report.outputfile = [path name]; set(gui.filename, 'String',param.report.outputfile);
    end
  end

  % initalization and save routines
  function initialize_callbacks()
    % pushbutton callbacks
    set(gui.okay, 'Callback', @okay_button); set(gui.cancel, 'Callback', @cancel_button);
    % filename selection callback
    set(gui.filename_sel, 'Callback', @filename_select);
  end

  function save_values()
    param.report.outputfile = get(gui.filename, 'String');
  end

  function initialize_values()
    set(gui.filename, 'String', param.report.outputfile);
  end

  function gui = get_gui_handles()
    gui.okay = findobj(hdlg, 'Tag', 'okay'); gui.cancel = findobj(hdlg, 'Tag', 'cancel');
    gui.filename = findobj(hdlg, 'Tag', 'filename');
    gui.filename_sel = findobj(hdlg, 'Tag', 'filename_select');
  end
end

% report generation
function param = generate_report(param)
  % generates report according to param.report

  % valid param.report.types:
  % csv_aggegrated - aggegrated list
```

```
  % csv_detailed – detailed list
  isdebug = isfield(param, 'debug'); file = fopen(param.report.outputfile, 'w');
  fprintf(file, 'm/c/f=%d/%f/%d\n', param.seg.pre_medfilt_len,...
  param.seg.pre_seg_thres, param.seg.pre_feat_size);
  if (param.seg.pre_tv)
    fprintf(file, 'alpha/N=%f/%d\n', param.seg.pre_tv_reg, param.seg.pre_tv_iter);
  end
  fprintf(file, 'file, cellnr, x, y\n'); N = length(param.files);
  for i = 1:N
    % get results filename
    curfile = param.files{i}; [pathstr, name, ext] = fileparts(curfile);
    resfile = fullfile(pathstr, [name '_res.mat']);
    % process segmented file
    if (exist(resfile, 'file'))
      data = load(resfile,'seg'); data = data.seg;
      found_cell_number = double(length(union(data(data  = 0),[])));
      [width_data height_data] = size(data);
      mean_density = double(100*found_cell_number/( width_data*height_data));
      fprintf(file, '[%s%s:_Found_cells: %d]\n', name, ext, found_cell_number);
      fprintf(file, '[%s%s:_Mean_density_per_10_x_10_px: %f]\n', name, ext, mean_density);
      mesg(isdebug, sprintf('Segmented data ''%s'' loaded.', resfile));
      cellval = unique(data(data ~= 0));
      [X,Y] = meshgrid(1:size(data,2), 1:size(data,1));
      for n = 1:length(cellval)
        massx = mean(X(data == cellval(n))); massy = mean(Y(data == cellval(n)));
        str = sprintf('%s%s,%d,%.2f,%.2f', name, ext, n, massx, massy); fprintf(file, [str '\n']);
      end
    else
      mesg(1,sprintf('Segmented data ''%s'' does not exist. Skipping.', resfile));
    end
  end
  fclose(file);
end
```

Last modification: 28.06.2013
